# Surgical Treatment of Patients with Lennox-Gastaut Syndrome Phenotype

**DOI:** 10.1100/2012/614263

**Published:** 2012-05-01

**Authors:** Shi-Yong Liu, Ning An, Xiang Fang, Prabhdeep Singh, Joseph Oommen, Qing Yin, Mei-Hua Yang, Yong Liu, Wei Liao, Chang-Qing Gao, Hui Yang

**Affiliations:** ^1^Department of Neurosurgery, Xinqiao Hospital, The Third Military Medical University, Chongqing 400037, China; ^2^Department of Neurology, University of Texas Medical Branch, Galveston, TX 77555, USA; ^3^Department of Rehabilitation, Southwest Hospital, The Third Military Medical University, Chongqing 400038, China; ^4^Department of Neurology, Xinqiao Hospital, The Third Military Medical University, Chongqing 400037, China; ^5^Department of Pediatrics, Xinqiao Hospital, The Third Military Medical University, Chongqing 400037, China; ^6^Research Center for Medical Sciences of The 3rd XiangYa Hospital, and The Center for Scientific Research with Animal Models, Central South University, Hunan, Changsha 410013, China

## Abstract

Lennox-Gastaut syndrome (LGS) is a devastating and refractory generalized epilepsy affecting children and adolescents. In this study we report the results of resective surgery in 18 patients with LGS phenotype who underwent single-lobe/lesionectomy or multilobe resection plus multiple subpial transection and/or callosotomy. After surgery, seven patients became completely seizure-free (Engel Class I) and five almost seizure-free (Engel Class II). Additional four had significant seizure control (Engel Class III), and two had no change in seizure frequency (Engel Class IV). Of the 4 patients without any lesion on brain MRI, 2 ended with Engel Class II, 1 with III and the other with IV in Engels' classification. Mean intelligence quotient (IQ) increased from 56.1 ± 8.1 (mean ± SD) before operation to 67.4 ± 8.2 (mean ± SD) after operation, a significant improvement (*P* = 0.001). Results also indicated that the younger the patient at surgery, or the shorter the interval between onset of seizure and resective operation, the better the intellectual outcome. Our data suggest that resective epilepsy surgery can be successful in patients with LGS phenotype as long as the EEG shows dominance of discharges in one hemisphere and corresponding ipsilateral imaging findings, even with contralateral ictal discharges.

## 1. Introduction

Lennox-Gastaut syndrome (LGS) is a generalized epilepsy characterized by an electroclinical trial of diffuse slow spikes-and-wave (SSW) complex with paroxysmal fast activity during sleep on electroencephalogram, mental retardation, and multiple types of generalized seizures, including atypical absences, tonic, and atonic seizures [1, 2]. LGS is usually caused by bilateral diffuse encephalopathy but localized cortical lesions, such as cortical dysplasia, cortical tuberous sclerosis, tumor, band heterotopia, and vascular malformation can also cause LGS [2–5]. In such cases, removal of cortical tumor or lesionectomy can result in seizure-free and normal development [6–9]. These observations suggest that LGS patients resulting from localized structural abnormalities are amenable to early surgical treatment with a significant impact on seizure control and cognitive development.

It has also long been noted that the interictal and ictal epileptic discharges in LGS patients are usually bilateral, synchronous, and symmetrical, but in some patients the SSW pattern shifts asymmetrically over the two hemispheres in different bursts, which suggests that multifocal lesions may exist in both hemispheres [10, 11]. Persistent focal or lateralized asymmetry of SSW activity may occur in as many as 25% of the LGS patients and is more common in patients with cognitive deficits [2, 11], which implies that focal lesions may exist in some subjects. Indeed, in sporadically reported cases multiple subpial transection with minimal cortical resection can result in satisfactory seizure control and/or IQ improvement in LGS subjects [6–8, 12]. Based on these observations, we believe that a considerable number of LGS patients may have a single- or multiple-unilateral cortical lesions causing secondary generalized epileptic discharges and that resective surgery should be effective in these patients.

Indeed, evidences have been accumulating to support our hypothesis. First, Wyllie and colleagues have demonstrated that children or adolescents with abundant generalized or bilateral epileptiform discharges on EEG can be successfully treated with surgery in selected patients [9]. Second, Lee and coworkers have recently showed that, despite abundant generalized and multiregional EEG anomalies, resective epilepsy surgery can be successful in some children with LGS [13]. However, in the study of Lee et al., patients with contralateral ictal epileptic discharges were all excluded. Here, we are the first ever to report, to the best of our knowledge, that epilepsy surgery can also be considered in children or adolescents with LGS phenotype with contralateral ictal discharges.

## 2. Methods

### 2.1. Diagnostic Criteria and Eligibility

In this retrospective chart study, we found that 52 patients during the period between 1997 and 2007 had LGS phenotype. Of them 18 patients underwent resective surgery and/or plus multiple-subpial transection (MST) and/or callosotomy. The defining characters of LGS used in the study were as follows: (1) multiple seizure types, mainly generalized, including tonic, atonic, and atypical absences; (2) primary and secondary SSW EEG discharges during wakefulness and paroxysmal fast activity (PFA) during sleep; (3) mental retardation.

Eligibility criteria for resective surgery included: (1) frequent (more than 4 times per month) and severe seizures interfering with the patient's life; (2) seizures refractory to at least two AEDs and surgery was considered to be the last resort after extensive discussion with the families; (3) focal or multifocal lesions confirmed by imaging data (MRI or SPECT or CT scan); (4) EEG showing ictal or interictal hemispheric-dominant discharges, that is, more than 70% of the discharges originating from one hemisphere in both ictal and interictal periods, a lateralizing abnormality which coincides with imaging or SPECT findings; (5) surgically accessible lesions, and the location of the lesions predicted that lesionectomy would cause no severe deficit; (6) parents' and/or patients' family had a reasonable understanding of the risks and benefits of the procedures. Duly-prepared informed consent forms were obtained. 

### 2.2. Preoperative Investigation

All patients in the study underwent a comprehensive evaluation including detailed history and neurological examination, routine and ambulatory EEG, long-term video EEG, and all satisfied the electroclinical criteria for LGS at least at some point in time. Some patients underwent magnetic resonance imaging (MRI), which was supplemented by ictal/interictal single-photon emission computed tomography (SPECT) scans and invasive intracranial monitoring with subdural plates and depth electrodes in some patients, when necessary. Protocol for interictal SPECT was as follows: thirty minutes after taking 400 mg potassium perchloric acid, patients were administered ^99m^Tc-ECD intravenously, and images were collected within 20 minutes. Procedures for ictal SPECT were as follows: on the day following the collection of the interictal images, patients were watched in the monitoring room; ^99m^Tc-ECD(0.4–0.5 MBq/Kg) was administered intravenously in 30 seconds as soon as seizures started or the typical epileptic discharges started, patients were scanned within 30 minutes of the attack. Image fusion was performed with CT data.

EEG dipoles were reconstructed and analyzed with MRI images using the Brain Electrode Source Analysis software (Version 5.1 beta, MEGIS Software GmbH; Germany). The primary epileptic foci were determined on the findings of these preoperative investigations.

### 2.3. Operative Procedures

Dense intraoperative electrocorticography (EcoG) was used to further confirm the localization of epileptogenic foci. Single-lobe resection or lesionectomy was performed when obvious epileptic discharge was found in one lobe or in a limited area, while multilobe resection in the same hemisphere was performed when multiple epileptogenic foci or diffuse discharges were confirmed. Callosotomy was performed in four patients with significant contralateral epileptic discharge or atonic seizure. Multiple subpial transection was undertaken when epileptic foci were found to be located in functional areas or remnant discharge was found after resective procedures. Resection of anterior temporal lobe was limited to within 5.0 cm posterior to the temporal pole in the dominant hemisphere and 5.5 cm in the nondominant hemisphere. The range for frontal lobe resection was from 3.0 to 6.0 cm posterior to the frontal pole, while that for occipital lobe resection was from 4.0 to 6.0 cm anterior to the occipital pole. Anterior callosotomy was performed from the genu to the tip of the hippocampal commissure, ranging in length from 4.0 to 7.0 cm (2/3 to 3/4 of corpus callosum), while the length of posterior callosotomy was 4.0–6.0 cm.

### 2.4. Intelligence Evaluation

The intelligence quotient (IQ) of children was tested using the Wechsler Preschool and Primary Scales of Intelligence (WPPSI) or Wechsler Intelligence Scale for Children-Revised (WISC-R), and adults were tested with a Wechsler Adult Intelligence Scale (WAIS-III). Memory was assessed using the Rey Auditory-Verbal Learning Test and the Rey-Osterrieth Complex Figure Test. Pre- and postoperative neuropsychological tests were performed by the same psychologist. Postoperative tests were repeated every 6 to 12 months during followup and the latest values were used for analysis.

### 2.5. Statistical Analysis

Paired *t*-tests were used to compare the pre- and postoperative scores of IQ, the Rey Auditory-Verbal Learning Test, and the Rey-Osterrieth Complex Figure Test. The Pearson correlation test was used to examine the relationship between IQ changes after operation, the interval between the onset of seizure and surgery, and the relationship between IQ changes after operation and the years of seizure. *P* < 0.05 was taken as statistically significant.

## 3. Results

### 3.1. Patient Demographics

18 patients, including 12 males and 6 females, aged 3–24 years (mean 11.5) at the time of surgery, underwent resective surgery. Preoperative characteristics of these subjects, including etiology, seizure type, EEG patterns, and imaging findings are shown in Table 1. Age at seizure onset ranged from 2 months to 6 years, with an average 3.5 years. The time period between epilepsy onset and surgery ranged from 2 to 21 years (average 8 years). Patients experienced, on average, 3.6 different seizure types prior to surgery. Patients had been using AEDs for a period of 1 to 19 years; the average number of AEDs tried was 4 and the average number of AEDs used was 2.8 at the time of surgery. Antiepileptic drugs were continued after surgery and gradually withdrawn if the patients were seizure-free and had marked improvement in EEG pattern. The follow-up period was between 1 to 9 years with a mean of 5.4 years.

 As shown in Table 1, four patients experienced pre- or perinatal hypoxic-ischemic insult; two cases had a history of head injury; two showed damage resulting from postnatal encephalitis; two, focal cortical dysplasia; and two, small vascular malformations. One was diagnosed with tuberous sclerosis. Five patients showed hemispheric or focal atrophy without a recognizable cause.

### 3.2. EEG and Image Findings

**Figure 1:**
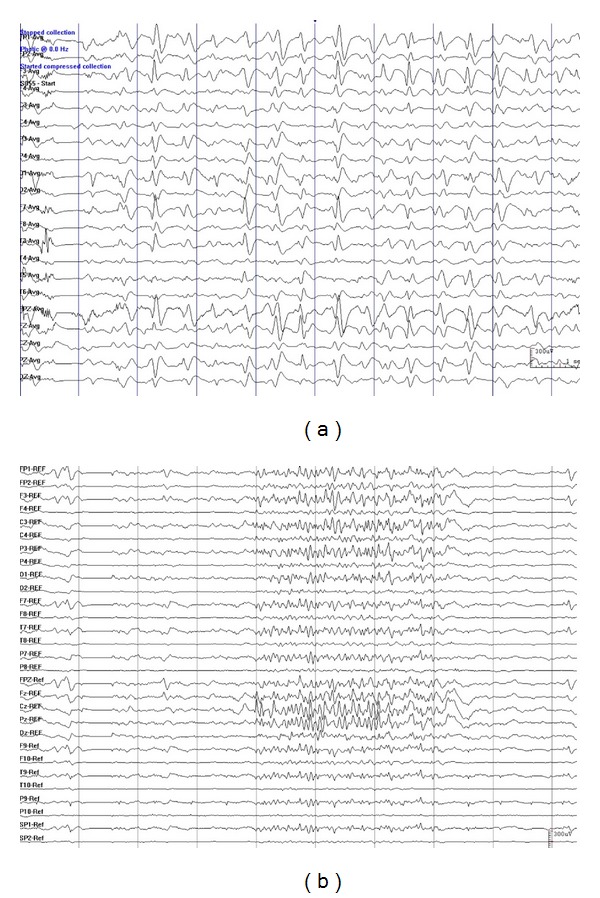
Representative EEGs showing slow spike-and-wave hemispheric dominance and PFA hemispheric dominance characterized by that the amplitude of epileptiform discharges in one hemisphere is higher than that of the other hemisphere (Figures 1(a) and 1(b)) and that the onset of the epileptiform discharges in one hemisphere is earlier than that of the other (Figures 1(a) and 1(b)), as well as that the duration of the epileptiform discharges in one hemisphere is shorter than that of the other (Figure 1(a)). Data were from patient no. 2 in Tables 1 and 2.

**Figure 2:**
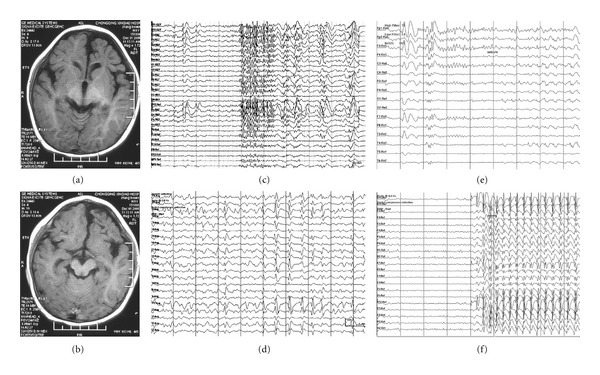
Clinical data of a patient with left frontal lobe atrophy. (a) and (b): MRI scan showing atrophy of left frontal lobe. (c) EEG showing PFA. (d) EEG showing SSW. (e) EEG showing epileptiform discharges during a tonic seizure. (f) EEG showing epileptiform discharges during an atypical absence. Data were from patient no. 13 in Tables 1 and 2.

**Figure 3:**
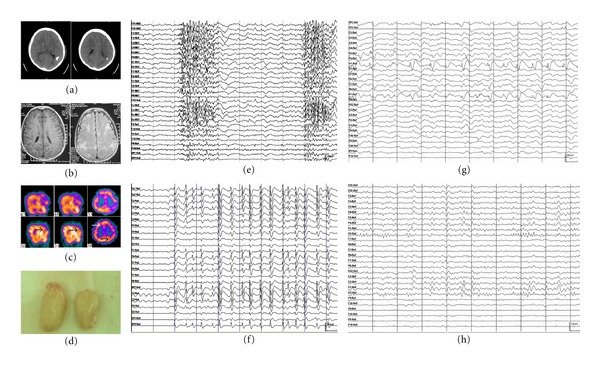
Clinical data of a patient with tuberous sclerosis. (a) CT scan showing calcification of the cortex and subependymal zone. (b) MRI showing subependymal zone tubers and cortex tubers. (c) Interictal (upper part) and ictal (lower part) SPECT results. Arrows point to interictal hypoperfusion and ictal hyperperfusion of the right frontal epileptic focus and left occipital epileptic focus. (d) Resected cortex tubers in operation. (e) EEG showing PFA. (f) EEG showing SSW. (g) EEG showing epileptiform discharges during a partial seizure (left occipital lobe). (h) Postoperative EEG. Data were from patient no.: 14 in Tables 1 and 2.

Results of routine EEG, and/or ambulatory EEG, and/or long-term video EEG monitoring were abnormal in all patients, and indicated an abnormal slow background activity/diffuse slowing. All patients showed symmetrical or asymmetrical 1.5 to 2.5 Hz SSW activity in the interictal EEG (primary bilateral synchrony in seven and secondary bilateral synchrony in 11 subjects) (Figures 1(a), 2(d), and 3(f)). PFA was noted in sleep EEGs in 17 patients (Figures 1(b), 2(c), and 3(e)). The epileptic discharges were characterized by bilateral symmetrical synchronous generalized discharge or bilateral asymmetrical discharge or bilateral asynchronous discharge or focal discharge. Hemispheric dominant discharges, that is, at least 70% of all discharges originating from one hemisphere, were noted in all patients in ictal and interictal EEGs. They showed the following additional characteristics: (1) asymmetrical discharge: the amplitude of epileptiform discharges in one hemisphere was higher than that of the other hemisphere (Figures 1(a), 1(b), 2(d), and 3(f)) or (2) asynchronous discharge A, the onset of the epileptiform discharges in one hemisphere was earlier than that of the other (Figures 1(a), 1(b), 2(e) and 2(f)); B, the duration of the epileptiform discharges in one hemisphere was shorter than that of the other hemisphere (Figure 1(a) or (3) Focal epileptic discharges (Figure 3(e)). Slow spikes-and-wave hemispheric dominance was seen in all 18 cases and PFA hemispheric dominance was seen in five cases. Two cases were found to have independent epileptic discharges in the contralateral hemisphere in ictal EEGs. However, these independent epileptic discharges accounted for less than 30% of the total epileptic discharges.

Eight out of the nine patients who underwent SPECT scan during both interictal and ictal periods showed typical blood flow changes, that is, interictal hypoperfusion and ictal hyperperfusion, whereas the other one showed interictal hypoperfusion and ictal normal perfusion. Four patients had only interictal SPECT scan, among them three had hypoperfusion and one had normal perfusion (Table 1). All of the 18 patients had MRI scans with 14 of them showing abnormalities (Table 1). Among the 4 patients with normal MRI, 3 of the patients had blood flow changes between ictal and interictal SPECT and 1 had head injury and his CT scan at the time of injury revealed abnormalities. The epileptic foci found by SPECT with the characteristics of blood flow changes were in agreement with those observed on EEG and neuroimaging in 10 of the 12 patients.

### 3.3. Surgical Procedures

The surgical procedure selected was determined on the comprehensive evaluation of the patients' clinical features plus the findings from EEG, MRI and SPECT. Three patients underwent single-lobe resection; among them one patient also underwent MST because of remnant discharges. Different combinations of multilobe resection were conducted in 15 patients as indicated by their preoperative assessments and EcoG. Eleven patients underwent frontal lobe resection; nine had temporal lobe resection; five had occipital lobe resection, and nine had focal cortical resection. Seven of the patients had two lobes resected, six, three lobes resected, and two, three lobes plus focal cortical resection. Four patients received partial corpus callosotomy because of bilateral or contralateral epileptogenic discharges, and most of the patients who underwent multilobar resection also received MST because of remnant discharges originating from functional zone (Table 2).

### 3.4. Pathological Findings

Specific abnormal pathology was observed in eight patients, whereas ten patients had nonspecific gliosis, as shown in Table 2.

### 3.5. Seizure Outcome and other Clinical Improvements

According to Engel's criteria, seven patients (38.9%) became seizure-free (Engel Class I) and five (27.8%) were almost seizure-free (Engel Class II). An additional four patients (22.2%) had significant seizure control (Engel Class III) and two (11.1%) had no change in seizure frequency (Engel Class IV; Table 2). Two patients with focal cortical dysplasia demonstrated excellent outcomes (Engel Class I). Of the two patients with vascular malformation, one achieved Engel's Class I status, whereas the other one fell into Engel Class III. Two cases with independent contralateral epileptic discharges that accounted for less than 30% of the total epileptic discharges in ictal EEGs achieved Engel's Class I and II, respectively. The outcome of two patients with a history of postnatal encephalitis was unfavorable (Class III and Class IV). One patient had recurrent epilepsy at three years of followup with sporadic but similar seizures to those experienced before operation. Three cases began to experience new seizure types that were not seen before.

Of 6 patients who had difficulty in expressive language function before operation; 4 improved greatly after surgery, and also had significant seizure reduction. Five of nine patients with behavior problems had significant improvement after operation. Improvements in cognitive function (see details below) and quality of life were also reported by the patients and their families.

### 3.6. EEG Changes

Although the preoperative slow background activity and diffuse slowing remained unchanged in most of the patients, 12 patients with Engel Class I or II outcomes showed marked improvement in EEG. Among them, 6 patients were free of epileptic discharges, and 3 showed rare spikes or SSW activity. Among the 6 patients with Engel Class III or IV outcomes, 5 were found to have discharges arising from the contralateral hemisphere and the remaining one had discharges arising from bilateral hemispheres.

### 3.7. Changes in AEDs Use

One child was no longer taking any AEDs at followup and the remaining 17 patients were on average taking two AEDs (range 1–3).

### 3.8. Intellectual Outcome

Comprehensive pre- and postoperative intellectual assessments were conducted in 15 of the 18 patients. In the remaining three, this assessment was difficult to perform due to lack of cooperation, which precluded a full pre- and postoperative comparison. The postoperative assessment was performed between 9–48 months (22.2 months on average) after surgery. Mean IQ before surgery was 56.1 ± 8.1 (mean ± SD) and increased to 67.4 ± 8.2 (mean ± SD) at postoperative assessment, which indicated significant improvement (*P* = 0.001). Whereas all patients had abnormal intellectual function (IQ < 70) before operation, only nine showed no improvement after operation (Table 2).

The level of improvement of IQ after operation was inversely correlated with the patients' age at surgery, that is, the younger the patient at surgery, the higher the IQ improvement after operation (*P* = 0.013, *r* = −0.62). The same is true for the relationship between years of seizure and IQ, that is, the shorter the interval between onset of seizure and the resective operation, the better the intellectual outcome (*P* = 0.004, *r* = −0.69). Furthermore, memory function improved significantly after operation in delayed recall testing (Rey Auditory-Verbal Learning Test) and both the copy and delayed recall tests (Rey-Osterrieth Complex Figure Test) (Table 3).

### 3.9. Complications

In this series, there was no perioperative death or life-threatening complications. Five patients experienced fever after surgery that lasted less than one week. Temporary complications were observed in seven patients, including acute disconnection syndrome (two cases), partial aphasia (three cases), and contralateral partial hemiplegia (two cases, of them one patient had partial aphasia and hemiplegia at the same time). All patients recovered within three weeks. Five patients who underwent occipital pole resection showed contralateral hemianopia, and among them, one had contralateral hemianopia before surgery. All patients gradually adapted to their hemianopia.

## 4. Discussion

In this study we found that resective surgery could be effective in improving IQ as well as effecting seizure control in patients with LGS phenotype with or without MRI lesions as long as there was dominance of EEG discharges in one hemisphere, even with ictal contralateral discharges. The patients who were younger or had a shorter interval between the onset of seizure and the resective operation had better IQ improvement after operation.

In agreement with previous observations [10, 11], we found in the current study that asymmetrical SSW discharge patterns existed in nearly one-third of LGS patients. Further, we observed that patients with hemispheric dominant PFA had an ictal discharge pattern; this had not been reported previously.

 Wyllie and colleagues achieved successful outcomes of epilepsy surgery in patients with generalized epileptiform EEG discharges and an extensive unilateral or strongly asymmetric congenital or early-acquired epileptogenic lesion on brain MRI [9]. During the preparation of this manuscript, Lee et al. reported successful results of resective epileptic surgery in 27 LGS patients, with 4 patients having no abnormalities on MRI [13]. In the current paper, 4 patients without any brain lesions on MRI were also included. In agreement with the findings of Lee et al. the seizure control of these patients was somewhat inferior to those with abnormalities on brain MRI(50% versus 60.8%)[13]. In our series 2 achieved II; 1, III and another, IV in Engels' classification. These results suggest that resective epileptic surgery can be successful in patients without any brain MRI abnormality, though somewhat less favorable than in those with abnormality.

Contrary to Lee et al. [13], our study included patients with ictal contralateral discharges, as long as the epileptic discharges originating in one hemisphere were >70% of all dischargesand imaging findings lateralized to the same side. To the best of our knowledge, this is the first ever report of surgical results for such patients. In broadening the eligibility criteria, more patients will be able to benefit from surgery.

There were also differences in the pathological findings of Lee's and our series. In Lee's [13], twenty patients (74%) were found to have cortical malformations. In ours there were only two such cases; the most common pathological findings being nonspecific gliosis (55%), followed by hippocampal sclerosis and nonspecific gliosis (17%). Accordingly, the surgical strategies and the outcomes of the seizure control in the two series varied. In Lee's [13], 22% of the patients underwent hemispherectomy, whereas in our series, 20% underwent multilobar resection plus MST and corpus callosotomy. Hence, 59% of Lee's patients reached seizure freedom while in our series only 39% were seizure-free, with another 50% showing significant improvement (Engel's Class II and III).

Contrary to the observation of Lee et al. [13], who found no significant association between the outcome of surgery and age at seizure onset, or duration of epilepsy before surgery, our results are similar to the general experience of epilepsy surgery performed in infancy [14]. Although these results may need to be reexamined further to rule out the effect of other factors, such as test-retest effect, practice effect, disease severity, and maturation, it is believed that surgical intervention possibly is an important option for older children and adolescents, just as in younger LGS patients, to prevent irreversible deterioration [13, 15].

In addition, in our study, of the seven patients who achieved level I in Engel's classification, five had specific focal lesions, and two had nonspecific lesions. Further, of the two patients with hippocampal sclerosis (HS) and nonspecific gliosis, one achieved I, the other achieved III in Engel's classification. It seems that patients with specific or focal lesions had a better outcome than those with nonspecific lesions, and that HS did not affect the progress.

It should be noted that the favorable surgical outcomes in selected patients with LGS phenotype achieved by us and others [13] are in sharp contrast with that reported by Bladin, whose poor results may be due to his performance of single, and, thus, incomplete resection of the lesions [16].

## 5. Conclusion

Our data are the first ever, to the best of our knowledge, to show that resective epileptic surgery can be successful in selected patients with LGS phenotype with or without MRI lesions, as long as there are dominance of EEG discharges in one hemisphere, and corresponding ipsilateral imaging findings, even with contralateral ictal discharges. 

## Figures and Tables

**Table 1:** Patient profiles—number 1.

Number	Age at surgery (years)	Duration of seizure at surgery (years)	Drug resistant time (years)	Etiology	Seizure type	EEG (characteristic and dominant locations)	MRI	SPECT
1	5	3	2.2	Hypoxic ischemic insult	Tonic + atonic + atypical absence + SPS	SSW + PFA, rt.sz (frontal + temporal + occipital)	Focal atrophy (rt. frontal)	

2	7	6	3	Head injury (previous CT scan shows subarachnoid hemorrhage)	Tonic + myoclonic + GTCS + atypical absence	SSW + PFA, lt.sz (frontal + temporal + parietal + occipital)	Normal	Interictal hypoperfusion and ictal hyperperfusion (lt. temporal + occipital)

3	12	10	9	Brain atrophy (determined by MRI)	Tonic + atonic + absence + SPS	SSW + PFA, lt.sz (frontal + temporal + parietal)	lt. hemisphere atrophy	Interictal hypoperfusion (lt. frontal + temporal)

4	6	2.9	1.8	Cortical dysplasia	Tonic + GTCS + SPS	SSW + PFA, rt. Sz (temporal)	Focal cortical dysplasia (rt. temporal)	

5	7	2.8	2.6	Head injury (previous CT scan shows mild brain contusion)	Tonic + myoclonic + GTCS	SSW + PFA, lt.sz. (frontal + temporal + parietal)	Normal	

6	9	4	2.5	Hypoxic ischemic insult	Myoclonic + GTCS + atypical absence + CPS	SSW + PFA, rt.sz (frontal + temporal + occipital)	rt. hemisphere atrophy	Interictal hypoperfusion and ictal hyperperfusion (rt. frontal + occipital)

7	6	1.9	1.4	Cortical dysplasia	Tonic + atypical absence + SPS	SSW + PFA, lt.sz (frontal)	Focal cortical dysplasia (lt. frontal)	

8	10	6	5	Hypoxic ischemic insult	Tonic + atonic + atypical absence + CPS	SSW + PFA, rt.sz (frontal + temporal + parietal + occipital)	rt. hemisphere atrophy	Interictal hypoperfusion (rt. parietal + temporal)

9	9	6	6	Vascular malformation	Tonic + CPS	SSW + PFA, lt.sz (parietal)	small vascular malformation (lt parietal)	Interictal normal

10	13	9	9	Brain atrophy (determined by MRI)	Tonic + myoclonic + atypical absence + GTCS	SSW + PFA, rt.sz. (parietal + occipital)	focal atrophy (rt parietal)	Interictal hypoperfusion and ictal hyperperfusion (rt. occipital)

11	9	6	6	Vascular malformation	Tonic + myoclonic + CPS	SSW + PFA, lt.sz. (frontal + temporal + parietal)	Vascular malformation (lt parietal)	Interictal hypoperfusion and ictal hyperperfusion (lt. temporal + parietal)

12	11	8	7	Encephalitis	Tonic + atypical absence + GTCS + CPS	SSW + PFA, lt.sz. (parietal + occipital)	Normal	Interictal hypoperfusion and ictal hyperperfusion (lt. temporal)

13	8	4	3	Brain atrophy (determined by MRI)	Tonic + myoclonic + atypical absence	SSW + PFA, lt.sz. (frontal + parietal + occipital)	lt. focal atrophy (lt frontal)	

14	13	7	4	Tuberous sclerosis	Myoclonic + GTCS + atypical absence + SPS	SSW + PFA, It.sz (temporal + occipital)	Cortex tubers and subependymal nodules	Interictal hypoperfusion and ictal hyperperfusion (rt. frontal and lt occipital)

15	17	16	15	Encephalitis	Tonic + atonic + GTCS + CPS	SSW + PFA, rt.sz (frontal + temporal + parietal)	Normal	Interictal hypoperfusion and ictal hyperperfusion (rt. frontal)

16	24	21	19	Hypoxic ischemic insult	Tonic + myoclonic + GTCS + SPS	SSW + PFA, lt.sz (frontal temporal + parietal)	lt. focal atrophy and perforating ventricle malformation	Interictal hypoperfusion (lt. temporal), and no change in ictal SPECT

17	19	15	15	Brain atrophy (determined by MRI)	Tonic + atypical absence + GTCS + CPS	SSW, rt.sz. (temporal + parietal)	rt. hemisphere atrophy and HS	Interictal hypoperfusion and ictal hyperperfusion (rt. temporal)

18	22	16	12	Brain atrophy (determined by MRI)	Tonic + atypical absence + atonic + CPS	SSW + PFA, lt.sz. (frontal + temporal + parietal)	lt. HS and focal atrophy (temporal)	Interictal hypoperfusion (lt. frontal + temporal)

Abbreviations: CPS: complex partial seizure; EEG: electroencephalogram; HS: hippocampal sclerosis; GTCS: generalized tonic-clonic seizures; LGS: Lennox-Gastaut syndrome; lt.: left; MRI: magnetic resonance imaging; PFA: paroxysmal fast activity; rt.: right; SPECT: single-photon emission computed tomography; SPS: simple partial seizure; SSW: slow spike-and-wave; sz.: seizure.

**Table 2:** Patient profiles—number 2.

Number	IQ prior to surgery	IQ after surgery	Surgery types	Pathological results	Outcome/Engel class	Followup (years)
1	44	74	Multilobe resection + MST	Nonspecific gliosis	I	2
2	53	61	Multilobe resection + corpus callosotomy + MST	Nonspecific gliosis	II	5
3	43	46	Multilobe resection + MST	Nonspecific gliosis	IV	1
4	46	72	Temporal excision	Focal cortical dysplasia	I	7
5	Unable to assess		Multilobe resection + MST	Nonspecific gliosis	II	8
6	61	73	Multilobe resection + MST	Nonspecific gliosis	II	5
7	Unable to assess		Frontal resection	Focal cortical dysplasia	I	9
8	51	63	Multilobe resection + corpus callosotomy + MST	Nonspecific gliosis	II	6
9	65	68	Lesionectomy + MST	Vascular malformation	III	7
10	58	65	Multilobe resection + MST	Nonspecific gliosis	I	6
11	62	78	Multilobe resection + MST	Vascular malformation	I	5
12	Unable to assess		Multilobe resection + corpus callosotomy + MST	Nonspecific gliosis	IV	9
13	54	72	Multilobe resection + MST	Nonspecific gliosis	III	3
14	49	79	Multilobe resection + MST	Tuberous sclerosis	I	3
15	64	61	Multilobe resection + corpus callosotomy + MST	HS and nonspecific gliosis	III	7
16	62	65	Multilobe resection + MST	Nonspecific gliosis	II	4
17	61	68	Multilobe resection + MST	HS and nonspecific gliosis	I	5
18	68	66	Multilobe resection + MST	HS and nonspecific gliosis	III	6

**Table 3:** Pre- and postoperative intellectual assessment scores.

RAVL test (*N* = 12)	Preoperation (mean ± SD)	Postoperation (mean ± SD)
Immediate recall scores	22.1 ± 7.5	26.3 ± 8.1
Delayed recall scores	3.4 ± 1.2	5.2 ± 1.4*

ROCF test (*N* = 12)		

Copy scores	20.1 ± 4.1	23.3 ± 3.2*
Delayed recall scores	13.3 ± 2.8	17.1 ± 2.7*

HRB** (** *N* = 6**)**	0.49 ± 0.18	0.36 ± 0.19

*Significantly different as compared with the value of preoperation (*P* < 0.05).

RAVL: Rey Auditory-Verbal Learning Test. ROCF: Rey-Osterrieth Complex Figure Test. HRB: Halstead-Reitan Battery.
